# Reduced cardiovagal baroreflex sensitivity is associated with postural orthostatic tachycardia syndrome (POTS) and pain chronification in patients with headache

**DOI:** 10.3389/fnhum.2023.1068410

**Published:** 2023-03-13

**Authors:** Bridget R. Mueller, Carly Ray, Alyha Benitez, Jessica Robinson-Papp

**Affiliations:** Department of Neurology, Icahn School of Medicine at Mount Sinai, New York City, NY, United States

**Keywords:** orthostatic intolerance, baroreflex, autonomic nervous system, cardiac reflexes, dysautonomia

## Abstract

**Background:**

Non-cephalgic symptoms including orthostatic intolerance, fatigue, and cognitive impairment, are common in patients with chronic headache disorders and may result from alterations in the autonomic nervous system. However, little is known about the function of autonomic reflexes, which regulate cardiovascular homeostasis and cerebral perfusion in patients with headache.

**Methods:**

Autonomic function testing data from patients with headache collected between January 2018 and April 2022 was retrospectively analyzed. Through review of EMR we determined headache pain chronicity and patient self-report of orthostatic intolerance, fatigue, and cognitive impairment. Composite Autonomic Severity Score (CASS), CASS subscale scores, and cardiovagal and adrenergic baroreflex sensitivities were used to quantify autonomic reflex dysfunction. Descriptive analyses (Mann-Whitney-*U* or χ^2^, as appropriate) determined associations between autonomic reflex dysfunction and POTS as well as chronic headache. Binomial logistic regression adjusted for age and sex. Spearman’s rank correlation determined the association between the total CASS score and the number of painless symptoms reported by each participant.

**Results:**

We identified 34 patients meeting inclusion criteria, of whom there were 16 (47.0%) with orthostatic intolerance, 17 (50.0%) with fatigue, 11 (32.4%) with cognitive complaints, and 11 (32.4%) with Postural Orthostatic Tachycardia Syndrome (POTS). The majority of participants had migraine (*n* = 24, 70.6%), were female (*n* = 23, 67.6%) and had a chronic (>15 headache days in a month) headache disorder (*n* = 26, 76.5%). Reduced cardiovagal baroreflex sensitivity (BRS-V) independently predicted chronic headache [aOR: 18.59 (1.16, 297.05), *p* = 0.039] and POTS [aOR: 5.78 (1.0, 32.5), *p* = 0.047]. The total CASS was correlated with the total number of non-painful features in the expected direction (*r* = 0.46, *p* = 0.007).

**Conclusion:**

Abnormal autonomic reflexes may play an important role in pain chronification and the development of POTS in patients with headache.

## 1. Introduction

Postural Orthostatic Tachycardia Syndrome (POTS), a form of orthostatic intolerance, is common in patients with primary headache (Khurana and Eisenberg, 2011; Cook and Sandroni, 2018). In the transition from an acute episodic pain disorder to a chronic pain disorder, patients fail to return to their neurological baseline. Orthostatic intolerance and other non-painful features including fatigue, mood alterations, and impaired cognitive function, frequently become unremitting and cause significant disability (Spierings and van Hoof, 1997; Minen et al., 2016; Aurora and Brin, 2017; Karsan and Goadsby, 2021). Although there is evidence that these symptoms are more prevalent in chronic compared to episodic headache, Peres et al. (2002); Zwart et al. (2003); Zucca et al. (2020) the neurobiology underlying their association to pain chronification is not well understood.

In patients with neurodegenerative and neuroinflammatory disorders, there is evidence that several non-painful features are associated with abnormal autonomic reflexes. Autonomic reflexes rely on caudal central nervous system (CNS) neurocircuitry and peripheral components of the autonomic nervous system (ANS) to maintain cardiovascular homeostasis and cerebral perfusion [30]. In a study examining ANS function in patients with Parkinson’s disease, a negative correlation between fatigue severity and heart rate response to deep breathing (HRDB), a commonly measured autonomic reflex, was reported (Olivola et al., 2018). In addition, baroreflex dysfunction was demonstrated in patients with both Multiple Sclerosis (MS) (Adamec et al., 2013; Habek et al., 2017) and Alzheimer’s disease (AD) (Meel-van den Abeelen et al., 2013; Femminella et al., 2014). The baroreflex regulates the cardiovascular response to orthostasis and other perturbations in homeostasis (Ogoh et al., 2010; Purkayastha et al., 2018; Ogoh and Tarumi, 2019). The neuroanatomy of the baroreflex involves vagal afferents which synapse in the brainstem, vagal efferents which exit the brainstem to modulate heart rate, and adrenergic sympathetic efferents which exit the spinal cord to regulate the vasomotor reactivity of peripheral blood vessels (Kaufmann et al., 2020). In MS, baroreflex dysfunction has been posited to underly the high prevalence of syncope and Postural Orthostatic Tachycardia Syndrome (POTS) (Adamec et al., 2013; Habek et al., 2017). In AD, impaired blood pressure recovery following an orthostatic challenge predicted a faster rate of cognitive decline (de Heus et al., 2020). These findings indicate deficits in autonomic reflexes may lead to orthostatic intolerance, fatigue, and cognitive impairment.

In contrast to patients with neurodegenerative and inflammatory neurologic conditions, quantification of autonomic dysfunction in patients with headache has focused on examination of vasomotor reactivity (Mamontov et al., 2016), plasma catecholamine levels (Mosek et al., 1999), and resting heart rate variability, a measure that correlates to prefrontal cortical and limbic network activity. Only a few studies have utilized more comprehensive autonomic testing that probes autonomic reflexes. One small study compared patients with low frequency episodic migraine and healthy controls and showed that an autonomic reflex, the Valsalva ratio, a measure of cardiovagal activity, did not differ between the two groups (Cortelli et al, 1991). However, a larger study including both episodic and chronic headache disorders, demonstrated patients with headache had reduced HRDB compared to healthy controls (Martin et al., 1992). There have been no studies examining the relationship between autonomic reflexes and the presence of orthostatic intolerance, fatigue, and cognitive impairment in patients with headache.

We hypothesize that abnormal autonomic reflexes play a role in the development of non-painful, features *and* in the chronification of pain, explaining their significant overlap. Our rationale is as follows. First, there is evidence in humans and animals that the autonomic reflex critical to a normal cardiovascular response to standing, the baroreflex, is also involved in modulating pain sensitivity. Reduced baroreflex sensitivity (BRS) is associated not only with orthostatic intolerance, but with increased sensitivity to pain and is present in patients with chronic pain disorders including fibromyalgia and lower back pain (Steptoe and Sawada, 1989; Nosaka, 1996; Shoemaker and Goswami, 2015). The neural pathway underlying the relationship between the baroreflex and pain processing involves the nucleus tractus solitarus (NTS), a brain region with anti-nociceptive activity and the major vagal afferent relay station. Second, there is evidence that reduced BRS is associated with abnormal cerebral perfusion (Laosiripisan et al., 2015). Abnormal patterns of cerebral perfusion have been demonstrated in chronic migraine, Petolicchio et al. (2016); Bogdanov et al. (2019) chronic non-migraine headache (Gilkey et al., 1997; Karacay Ozkalayci et al., 2018), chronic fatigue syndrome (Staud et al., 2018; Li X. et al., 2021) and impaired cognition (Li R. et al., 2021; Coffin et al., 2022; Wang et al., 2022). Finally, a recent, small study examining skin biopsies of patients with migraine reported nearly 50% had evidence of small fiber neuropathy (Stillman et al., 2021). While the mechanism of small fiber neuropathy in patients with headache is unclear, sympathetic denervation impairs the vasomotor response to orthostasis and is present in neuropathic POTS (Stillman et al., 2021). These findings raise interesting questions regarding the role of the peripheral ANS in the development of chronic pain, as well as the non-painful features which commonly accompany it including orthostatic intolerance, fatigue, and impaired cognition.

We conducted a retrospective chart review of consecutive patients with headache who presented for autonomic function testing (AFTs) at a single center. We included both migraine and non-migraine headache syndromes, as we anticipated that the caudal CNS and peripheral neural circuitry underlying autonomic reflexes would be agnostic to the specific headache diagnosis. Through review of electronic medical records (EMR) we determined headache pain chronicity and patient self-report of non-painful features including orthostatic intolerance, fatigue, and cognitive impairment. Our overarching hypothesis was that autonomic reflex abnormalities underlie both chronification of headache pain and the development of non-painful features, accounting for their frequent co-occurrence. Specifically, we sought to investigate whether autonomic reflex dysfunction, as quantified by the validated Composite Autonomic Severity Score (CASS) and BRS would be: (1) more common in patients with chronic as opposed to episodic headache, and (2) correlated with the presence of orthostatic intolerance and other non-painful features.

## 2. Materials and methods

### 2.1. Study design and sample

We performed a retrospective chart review of patients with headache who received a full battery of autonomic function testing (AFT) at the Mount Sinai Autonomic Laboratory between January 2018 and April 2022. Due to the COVID-19 pandemic, the laboratory was not testing patients from March 2020 through June 2020. Further, the Valsalva Maneuver, an aerosolizing procedure, was not performed during July 2020-April 2021 and November 2021-February 2022, when COVID-19 transmission rates were high and non-essential aerosolizing procedures were prohibited by our institution. The sample size was determined by the number of patients meeting eligibility requirements during the study period.

Using a consecutive approach, we retrospectively chart-checked all patients who received autonomic testing during the stated time periods, using *International Classification of Diseases, Tenth Revision, Clinical Modification* (ICD10) codes, review of clinical documentation, and referral notes to identify patients with headache. Eligibility criteria included a diagnosis of headache for more than 6 months prior to autonomic testing, clinical documentation of headache characteristics, and an age between 15 and 75 years. Exclusion criteria included COVID-19 infection within the preceding 6 months of autonomic testing, a medical condition associated with dysfunction of the ANS (e.g., diabetes, Parkinson’s disease), and unclear documentation of headache frequency and semiology. In addition, patients taking medications that impacted cardiovascular function (e.g., beta-blockers or angiotensin-receptor II blockers) or had a significant anti-cholinergic burden (e.g., tricyclic antidepressant at doses higher than 20 mg per day) were excluded. The Mount Sinai Hospital Institutional Review Board approved the use of patient data for this retrospective study and waived the requirement for informed consent.

### 2.2. Data collection and characterization of study sample

Clinical documentation and autonomic function testing referrals in our institution’s electronic health record system (Epic, Verona, WI) were retrospectively reviewed to obtain patient’s self-reported sex, age at time of autonomic testing, average number of headache days per month, and presence of fatigue, cognitive complaints, and orthostatic intolerance.

Headache diagnoses were obtained through ICD10 codes used for visit billing and confirmed or clarified through review of clinical documentation and correspondence with referring headache physician or neurologist. Headache syndromes were classified based on the International Classification of Headache Disorders, Third Edition (ICHD-3). Both migraine and non-migraine headache disorders were eligible. Patients were characterized as having a chronic headache disorder if they reported an average of 15 or more headache days per month during the three months preceding autonomic testing. If patients reported an average headache frequency < 15 days per month during the three months prior to testing, they were characterized as having an episodic headache disorder. Patients were classified as having fatigue if a provider documented in notes or billing codes the presence of “lethargy,” “fatigue,” or “excessive sleepiness.” A patient was characterized as having cognitive impairment if a provider documented in notes or billing, impaired “attention,” “focus,” “memory,” or “brain fog.” A patient was characterized as having orthostatic intolerance if a provider documented in notes or billing codes the presence of orthostatic “dizziness” or “lightheadedness.” In addition, elements of the autonomic testing were used to confirm orthostatic intolerance according to standard criteria for Postural Orthostatic Tachycardia Syndrome (POTS) which are a symptomatic sustained increase in heart rate ≥ 30 beats per minute for adults, and ≥40 beats per minute for patients 15–19 years of age during tilt upward compared to supine baseline in the absence of orthostatic hypotension. The upright portion of the tilt table testing is 10 min. The number of concurrent non-painful, features (fatigue, orthostatic intolerance, and cognitive impairment) was also summed for each patient (from 0 to 3).

### 2.3. Clinical autonomic nervous system assessment

A standard battery of autonomic function tests (AFTs), as previously described, Low (1992); Low and Opfer-Gehrking (1999) including quantitative sudomotor axon reflex testing (QSART), heart rate response to deep breathing (HRDB), Valsalva maneuver (VM), and tilt table testing was performed for all patients at our center. Testing equipment and data analysis software are supplied by WR Medical Electronics Co., (Maplewood, MN). The QSART was performed by placing a capsule containing acetyl choline (ACh) on the skin in four standardized locations (forearm, proximal leg, distal leg, and foot). The capsule was attached to an automated system which delivers a small continuous electrical stimulus to the capsule causing iontophoresis of ACh into the skin, which triggers a reflexive sweat response collected by the capsule. The total sweat volume was measured and compared to standardized values. Second, a non-invasive continuous beat-to-beat blood pressure monitoring device was attached to the participant’s finger (Nexfin system^1^) and a 3-lead surface electrocardiogram attached to the chest. Continuous blood pressure (BP) and heart rate (HR) were recorded during the VM (forced exhalation to a pressure of 40 mmHg for 15 s) and during a standardized paced deep breathing exercise (HRDB). Finally, the participant rested in the supine position for 5 min before being secured to the tilt table and tilted upright 75 degrees. Continuous HR and BP and orthostatic symptoms were recorded for 10 min while the patient was upright.

### 2.4. Description of autonomic nervous system measures

Continuous HR and BP were recorded, and the raw data stored during the VM, HRDB and tilt table testing. Using visual inspection of graphically represented HR and BP data combined with functionality in the WR Testworks software, two markers of reflexive HRV are calculated. The Valsalva ratio (VR) is calculated by dividing the highest HR during the VM to the lowest HR immediately following the maneuver. During the deep breathing task, the HR rises and falls in rhythm with the breath. For HRDB, the average change in HR from peak inspiration to expiration is calculated for the five consecutive cycles of breath which yield the largest result.

The Composite Autonomic Severity Score (CASS) is an age- and sex-adjusted summary score which provides an overall measure of autonomic reflexive function and sudomotor, vagal, and adrenergic sub-scores [9]. The sudomotor sub-score uses data from the QSART, the vagal sub-score is based on the VR and HRDB, and the adrenergic sub-score is based on BP changes during VM and tilt table testing. The authors of the CASS suggest that a CASS < 2 is normal, and that a CASS of 2–3 represents mild autonomic neuropathy (Low et al., 2003). We chose a more stringent threshold of a CASS ≥ 3.

Baroreflex sensitivity (BRS) was calculated as previously described (Palamarchuk et al., 2014). Briefly, the Valsalva maneuver data is visually inspected by a trained, blinded technician. BRS-V is a measure of the compensatory cardiac response to a decrease in BP evoked during the forced expiration against a closed glottis and is calculated by dividing the change in RR interval during phase 2E of the VM by the change in systolic blood pressure. It is expressed as milliseconds/mmHg. BRS-A, reflective of primarily beta-1 adrenergic activity is expressed in mmHg/S and it is calculated by dividing the change in systolic blood pressure during phase 3 by the time required for SBP to recover following the release of VM Normal values of BRS-V and BRS-A have been established, as well as suggested groupings of abnormal values into five (BRS-V) and three (BRS-A) ascending categories (Low, 1993).

### 2.5. Statistical analysis

Descriptive statistics were performed for demographic and clinical variables. We used the Mann-Whitney *U* test to compare continuous variables and the Chi-square analysis to compare categorical variables, respectively, between chronic and episodic headache groups as well as migraine and non-migraine headache, without correction for multiple comparisons. While CASS normative scoring is adjusted for age and sex, BRS is not. Therefore, multivariate logistic regression adjusting for age and sex was performed as appropriate for analyses involving BRS. Finally, Spearman’s rank correlation was performed to determine the association between the total CASS score and the number of non-painful features. All analyses were two-tailed and conducted at the alpha = 0.05 level using SPSS version 24.

## 3. Results

### 3.1. Participants

Our sample (*n* = 34) had an average age of 26 years and 67.6% were female (Table 1). The majority of participants had migraine (70.6%) and satisfied criteria for a chronic headache disorder (76.5%). Supplementary Table 1 lists the *N* of each headache disorder. The self-reported average monthly headache frequency ranged from 1 to 30 days. For participants with episodic headache, the median was 3 headache days per month, while for those with chronic headache, the median was 30 headache days per month. Most participants were relatively recently diagnosed, with a median disease duration of 5 years (IQR, 1.0, 12.0) Four patients were excluded due to insufficient documentation of headache frequency and characteristics. Two patients were excluded due to taking amitriptyline at a dose greater than 20 mg per day. There was no information regarding disease duration for four participants.

**TABLE 1:** Patient characteristics*.

	Overall *N* = 34	Episodic *N* = 8	Chronic *N* = 26	*p*-value
Sex (Female)	23 (67.6)	3 (37.5)	20 (76.9)	0.079
Age at time of testing [median (1q, 3q)]	26.0 [18.0, 37.5]	40.5 [28, 53]	26.0 [21, 39]	0.039
Duration of headache syndrome (years) [median (1q, 3q)]	5.0 [1.0, 12.0]	9.5 [5.0, 13.0]	5.0 [2.1, 15.0]	0.038
Headache days per month [median (1q, 3q)]	30.0 [13.0, 30.0]	3.0 [1.4, 7.0]	30.0 [25.0, 30.0]	<0.001
Daily headache	19 (55.8)	0 (0.0)	19 (73.1)	<0.001
Report of a non-painful feature	19 (55.8)	2 (25)	17 (65.4)	0.044
Fatigue	17 (50.0)	1 (12.5)	16 (94.1)	0.039
Orthostatic intolerance	14 (41.4)	1 (35)	13 (61.5)	0.611
Postural orthostatic tachycardia syndrome (POTS)	11 (32.3)	1 (12.5)	10 (38.5)	0.176
Cognitive complaint	11 (32.3)	2 (25)	9 (34.6)	0.170
Number of painless symptoms (median, 1q, 3q)	1.0 [0.0,2.0]	0.0 [0.0,0.75]	1.0 [0.0,3.0]	0.031
Taking headache prevention medication	20 (58.8)	3 (37.5)	18 (69.2)	0.124

*Unless noted, dated is presented as *N* (% column).

The majority of participants had at least one of the non-painful features (55.8%, Table 1) with approximately half reporting fatigue (50.0%), while nearly one-third reported cognitive complaints (32.3%) and/or orthostatic intolerance (32.3%). The majority (58.8%) of participants reported taking a daily medication to prevent headache at the time of autonomic testing. See Supplementary Table 2 for list of medication classes.

### 3.2. Description of autonomic measures

Abnormal CASS scores were prevalent with 15 (44.1%) participants meeting criteria for autonomic dysfunction (CASS ≥ 3). Abnormalities were seen in all three autonomic sub-scores (cardiovagal, adrenergic, and sudomotor), but were most common in the sudomotor score. Ninety-three percent of participants meeting criteria for autonomic dysfunction had sudomotor deficits, with all levels of severity represented (score of 1–3). Adrenergic deficits were seen in 66.7% of participants with autonomic dysfunction, but nearly all were at the lowest severity level (1 out of 4) with only three participants scoring a 2 out of 4. Cardiovagal abnormalities were present in 46.7% of participants with autonomic dysfunction, again nearly all at the lowest severity level (score 1 out of 3). Eighty percent of patients with autonomic dysfunction had deficits in two sub-scores and 20% had deficits in all three. An abnormal BRS-V was present in 47.1% of patients, but the majority (82.2%) of deficits were mild or moderate (score 1–3 out of 5). Reduced BRS-A was present in 88.2% of patients, and again, the majority of deficits were mild and moderate (score of 1–2) with only three participants scoring a 3 out of 3. Autonomic indices did not differ between migraine and non-migraine headache disorders (*p* > 0.05 for all comparisons. See Supplementary Table 3).

### 3.3. Clinical characteristics and autonomic reflexes in participants with episodic versus chronic headache

Duration of headache syndrome in years did not differ between patients with chronic and episodic headache. However, compared to patients with episodic headache, those with chronic headache were younger when headaches began. Non-painful features were more common in patients with chronic headache compared to episodic headache (Table 1).

**FIGURE 1:**
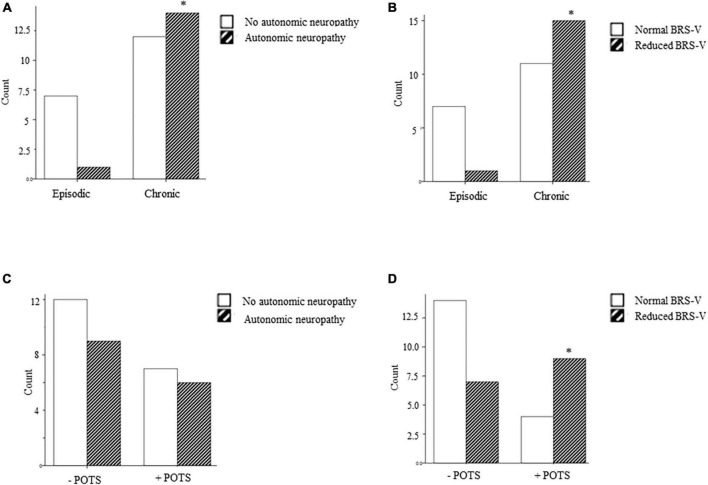
**(A–D)** Frequency of autonomic dysfunction (CASS ≥ 3), and reduced cardiovagal baroreflex sensitivity (BRS-V) in patients with episodic and chronic headache and with and without POTS. * = main effect, *p* < 0.05.

Participants with chronic headache had a significantly higher total CASS compared to patients with episodic headache and a greater proportion of participants with chronic headaches met criteria for autonomic dysfunction (58.3%) compared to those with episodic headache (12.5%) (Figure 1 and Table 2). In patients with chronic headache, deficits in adrenergic and sudomotor function were most common. Reduced BRS-V was also more common in those with chronic headache (57.7%) compared to episodic (12.5%). This association remained significant in multivariate regression [aOR: 18.6 (1.2, 297.0), *p* = 0.039]. The proportion of participants with abnormal BRS-A scores did not differ between those with chronic and episodic headache disorders.

**TABLE 2:** Associations between autonomic reflexes, headache chronicity*.

	Overall *N* = 34	Episodic *N* = 8	Chronic *N* = 26	*p*-value
Total CASS	2.0 [0.0, 4.0]	0.5 [0.0, 6.7]	3.0 [1.0, 5.0]	0.004
Autonomic dysfunction (CASS ≥ 3), *N* (% column)	15 (44.1)	1 (12.5)	14 (53.8)	0.039
Sudomotor CASS	1.0 [0, 2.25]	0.0 [0.0, 0.0]	2.0 [0.0, 5.0]	0.006
Adrenergic CASS	1.0 [0.0, 1.0]	0.0 [0.0, 0.0]	1.0 [0.0, 2.0]	0.006
Cardiovagal CASS	0.0 [0.0, 1.0]	0.0 [0.0, 1.0]	0.0[0.0, 1.0]	0.823
BRS-V, ms/mmHg [abnormal, *N* (% column)]	16 (47.1)	1 (12.5)	15 (57.7)	0.025
BRS-A, ms/mmHg [abnormal, *N* (% column)]	30 (88.2)	8 (100.0)	22 (84.6)	0.551

*Unless otherwise noted, data presented is median (1q, 3q). BRS-A, adrenergic baroreflex sensitivity; BRS-V, vagal baroreflex sensitivity.

### 3.4. Association of autonomic reflexes with non-painful features (orthostatic intolerance, fatigue, and cognitive impairment)

The total CASS was correlated with the total number of non-painful features in the expected direction (*r* = 0.46, *p* = 0.007). A reduced BRS-V was also significantly associated with the report of at least one non-painful feature (*p* = 0.002). Reduced BRS-V was demonstrated in 63% (12/19) of participants reporting at least one non-painful feature and 82% (9/11) of participants reporting at least two. There was no correlation between BRS-A and non-painful features.

With regard to orthostatic intolerance specifically, neither CASS scores nor BRS-A categorization differed between participants who did or did not meet criteria for POTS (*p* > 0.05). However, a greater proportion of participants with POTS had an abnormally reduced BRS-V compared to those without POTS (72.7 versus 34.8% *p* = 0.038). In multivariate regression, BRS-V maintained its significant association with POTS [adjusted odds ratio, aOR: 5.78 (1.0, 32.5), *p* = 0.047].

## 4. Discussion

In this study examining autonomic function in patients with headache, we found that autonomic dysfunction was common. Consistent with our hypothesis, the prevalence and severity of autonomic reflex dysfunction was greater in patients with chronic headache but did not differ between migraine and non-migraine headache disorders. In addition, we demonstrated that the cardiovagal component of baroreflex sensitivity (BRS-V) was associated with both POTS and chronic headache. Finally, we showed increasing severity of autonomic reflex dysfunction correlated with the number of non-painful features (orthostatic intolerance, cognitive impairment, fatigue) over the entire sample. Together, these novel findings suggest that dysfunction of autonomic reflexes should be investigated further as a potential mechanism underlying pain chronification and the development of non-painful features in people with chronic pain conditions.

Our results demonstrate that autonomic dysfunction in patients with chronic headache and non-painful features is widespread and involves abnormal baroreflex, adrenergic, and sudomotor function. As we hypothesized, function of autonomic reflexes did not differ between patients with migraine and non-migraine headache syndromes. Deficits in adrenergic function and the sudomotor reflex, which relies on post-ganglionic sympathetic cholinergic efferents, were most prominent and suggest significant pathology in the sympathetic nervous system (SNS). The SNS is a physiologically diverse and complex system with an extensive network of efferent projections to all organ systems. Future studies should examine the function of post-ganglionic sympathetic efferents to other organ systems in patients with headache.

**FIGURE 2:**
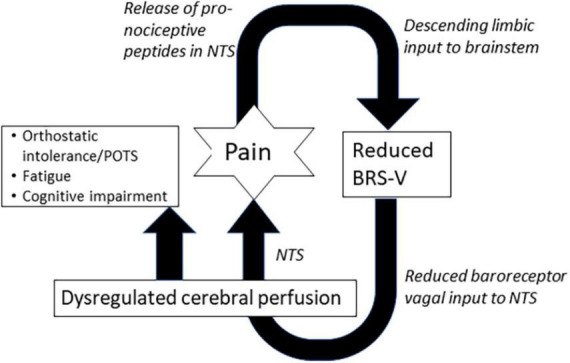
Positive feed-forward loop illustrating potential connections between pain, cardiovagal baroreflex sensitivity (BRS-V), and non-painful features. NTS, nucleus tractus solitarus.

Our findings demonstrate that adrenergic baroreflex sensitivity (BRS-A) was frequently diminished in all our patients but did not distinguish those with episodic versus chronic headache. In contrast, a greater proportion of patients with chronic headache had reduced BRS-V compared to episodic headache. Several possible mechanisms could explain the relationship between BRS-V and chronic headache. Reduced baroreceptor vagal afferent input to the nucleus tractus solitarus (NTS), a brain region with anti-nociceptive activity (Oley et al., 1982; Blomqvist et al., 2000; Pickering et al., 2003) may lead to hyperalgesia and vulnerability to chronic pain. In addition, deficits in BRS-V are associated with reduced cerebral perfusion and hypoperfusion is a powerful promoter of neuroinflammation and gliosis, two pathologies strongly linked to numerous headache disorders (Laosiripisan et al., 2015). Finally, there is evidence in animals and humans that that nociception suppresses the baroreflex through both top-down (Nosaka, 1996) and bottom-up processes (Pickering et al., 2003) to optimize fight and flight activities. The amygdala and hypothalamus are higher order brain regions involved in the suppression of baroreflex during pain (Nosaka, 1996). There is also evidence in animals that noxious stimulation leads to an increase in substance P in the NTS and a suppression of BRS-V, but not BRS-A (Pickering et al., 2003). Therefore, a feed-forward loop may exist (Figure 2.) An initial acute episode of nociception that suppresses BRS, may reduce cerebral perfusion and/or result in decreased activity in anti-nociceptive brain regions, increasing vulnerability to future painful episodes, which further decreases BRS (Figure 2). Interestingly, while the duration of headache illness did not differ between patients with episodic and chronic illness, patients with chronic headache were significantly younger at the time of headache onset compared to patients with episodic headache. Therefore, it is possible that developmental windows exist when the neural circuitry of the baroreflex is particularly vulnerable to long-term suppression by painful experiences.

We also found reduced BRS-V was present in a greater proportion of patients who reported a non-painful feature, and more broadly, that the severity of autonomic dysfunction, as measured by the total CASS score correlated to the number of painless symptoms in patients with headache. Participants generally reported more than one non-painful symptom. Thus, autonomic phenotyping of individual non-painful features was not feasible. However, BRS-V was an independent predictor of POTS, a disorder that is characterized not only by orthostatic intolerance, but fatigue and cognitive impairment (Cook and Sandroni, 2018). Cardiovascular exercise, an important treatment for POTS has been shown to increase baroreflex sensitivity.

We propose three potential mechanisms connecting abnormal reflexes to fatigue and cognitive impairment to be tested in future studies. The first is reduced or dysregulated cerebral perfusion. Abnormal baroreflex function and sympathetic denervation of the vasculature may both impair the regulation of cerebral perfusion (Asil et al., 2007; Liau et al., 2008; Nasr et al., 2011), which has been demonstrated in chronic fatigue syndrome, Staud et al. (2018); Li X. et al. (2021) and in patients with cognitive impairment (Li R. et al., 2021; Coffin et al., 2022; Wang et al., 2022). Second, reduced vagal afferent activity from baroreceptors has been associated with changes in the sleep/wake cycles (Tsai et al., 2021; Karemaker, 2022) day-time sleepiness (Peckerman et al., 2003) and reduced performance in cognitive tasks (Reyes del Paso et al., 2004; Paso et al., 2012; Herman and Tsakiris, 2021). There is evidence that this occurs through bottom-up processing, as vagal afferent input to the NTS may alter higher cortical networks (Kerfoot et al., 2008; Garcia et al., 2017). Finally, inflammation may link abnormal autonomic reflexes to fatigue and cognitive impairment, as well as headache chronification. Deficits in both adrenergic and sudomotor CASS sub-scores suggest the presence of post-ganglionic sympathetic denervation. The loss of sympathetic efferents is associated with decreased anti-inflammatory action at local β-adrenergic receptors and increased activity at systemic, pro-inflammatory α-adrenergic receptors. Sympathetic denervation is specifically associated with higher levels of pro-inflammatory tumor necrosis factor-α (TNF-α) and interleukin-6 (IL-6) (Schneemilch and Bank, 2001). Elevations in proinflammatory cytokines, including IL-6, have been demonstrated in patients with orthostatic intolerance (Okamoto et al., 2015), fatigue (Montoya et al., 2017) and cognitive impairment (Flores-Aguilar et al., 2021). Sensory sensitization, a process closely linked to headache chronification (Scheuren et al., 2020) and POTS, Bigal et al. (2008); Cortez et al. (2021) may also result from increased levels of pro-inflammatory cytokines (Kawasaki et al., 2008).

Our study has several limitations. First, the presence of non-painful features was determined by patient self-report to a clinical provider, not a standardized questionnaire. Therefore, severity, frequency, and duration of fatigue, orthostatic intolerance, and cognitive impairment could not be assessed. Second, patients are typically referred for autonomic testing by a physician to evaluate a symptom associated with ANS dysfunction. Therefore, the prevalence of autonomic symptoms and POTS in our sample may not be generalizable and larger prospective studies are needed. Relatedly, our data suggest that patients with episodic headaches are less likely to present with the non-painful symptoms of headache associated with ANS dysfunction that may prompt a referral for autonomic testing. Therefore, our study sample did not contain a sufficient number of patients with episodic headache to permit correction for multiple comparisons and the significance of the univariate analyses and regression analyses should be interpreted with caution. Future prospective studies are planned to address this limitation. Finally, additional confirmatory markers of autonomic dysfunction such as intraepidermal nerve fiber density on skin biopsy or catecholamine plasma levels in response to standing, were not obtained. In an acknowledgment of this limitation, we chose a higher CASS threshold to identify autonomic dysfunction.

In conclusion, our findings demonstrate that reduced cardiovagal baroreflex sensitivity was associated with the presence of non-painful features including orthostatic intolerance, fatigue, and cognitive impairment, as well as pain chronification in patients with headache. In addition, we found deficits in autonomic reflexes that suggested the presence of widespread pathology of the sympathetic nervous system and highlight the importance of the peripheral nervous system in leading to both orthostatic disorders and chronic pain. Future studies should probe potential mechanisms linking autonomic reflexes to the development of non-painful features and pain chronification by measuring cerebral perfusion and markers of inflammation in patients with chronic pain.

## Data availability statement

The original contributions presented in this study are included in the article/Supplementary material, further inquiries can be directed to the corresponding author.

## Ethics statement

The studies involving human participants were reviewed and approved by Icahn School of Medicine at Mount Sinai Hospital. Written informed consent for participation was not required for this study in accordance with the national legislation and the institutional requirements.

## Author contributions

BM drafted the manuscript and analyzed the data. CR performed the chart review. AB computed autonomic measurements including the Composite Autonomic Severity Score (CASS). JR-P contributed to the design and edited the manuscript. All authors contributed to the article and approved the submitted version.

## Footnotes

1
www.bmeye.com
