# Supplementary material for: Reduced cardiovagal baroreflex sensitivity is associated with postural orthostatic tachycardia syndrome (POTS) and pain chronification in patients with headache
Source: Front Hum Neurosci. 2023 Mar 13;17:1068410. doi: 10.3389/fnhum.2023.1068410 (PMC10040804; doi:10.3389/fnhum.2023.1068410)
Supplement: Supplementary file 1 [file Table_1.DOCX]

**SUPPLEMENTAL TABLES**

| Table 1: Headache Diagnoses | N = 34 | |
| --- | --- | --- |
| Migraine | | 24 (70.6) |
| Migraine without aura | | 18 (52.9) |
| Migraine with aura | | 6 (17.6) |
| Non-migraine | | 10 (29.4) |
| Tension-type | | 4 (11.8) |
| Low pressure | | 2 (5.9) |
| Cervicogenic | | 2 (5.9) |
| Post-traumatic | | 2 (8.8) |
| Chronic headache syndrome (cephalgia > 15 days/month) | | 26 (76.5) |
| Episodic headache syndrome | | 8 (23.5) |

| Table 2: Headache Prevention Treatment | N = 20 |
| --- | --- |
| Topiramate | 8 |
| Gabapentin | 3 |
| Venlfaxine | 4 |
| Amitriptyline (< 20 mg daily) | 2 |
| Magnesium | 1 |
| Botox and galcanezumab | 1 |
| Singulair, doxycycline, and naltrexone | 1 |

| Table 3: Autonomic indices compared between migraine and non-migraine headache disorders | | | |
| --- | --- | --- | --- |
|  | Migraine  N = 24 | Non-migraine  N= 10 | p-value |
| CASS total  *[median, IQR]* | 2.0 [0.0,4.0] | 2.5  [0.0, 2.0] | 0.564 |
| Sudomotor CASS *[median, IQR]* | 1.5  [-0.5,3.5] | 1.0 [0.0,4.0] | 0.669 |
| Adrenergic CASS *[median, IQR]* | 0.5  [0,1.5] | 1.0  [0.0, 2.0] | 0.838 |
| Cardiovagal CASS *[median, IQR]* | 0.0  [0.0, 1.0] | 0.0  [0.0, 1.0] | 0.615 |
| Diagnostic Criteria for POTS satisfied | 10 (40.0) | 1 (10.0) | 0.072 |
| BRS-V, ms/mmHg  < 4 (abnormal) | 12 (50.0 ) | 4 (40.0) | 0.595 |
| BRS-A ms/mmHg  *median, [1q,3q]* | 16.0 [6.0,26.0] | 14.0  [4.0,24.0] | 0.592 |
| Baseline SBP | 119 [99,139] | 120 [99,141] | 0.528 |
| Baseline DBP | 72 [65,79] | 72[62,82] | 0.867 |
| Drop in BP during phase IIE | 10.0 [4,16] | 12.0 [6,20] | 0.894 |
| Note: IQR: interquartile range. BRS-A: adrenergic baroreflex sensitivity, BRS-V; vagal baroreflex sensitivity, BRS-V | | | |
